# Performance of elderly on the three words-three shapes test: a Brazilian study

**DOI:** 10.1590/S1980-57642008DN10400012

**Published:** 2007

**Authors:** Cristiane Garcia da Costa Armentano, Julieta Quayle

**Affiliations:** 1 Psychologist. Specialist in Neuropsychology from the Center of Psycho-Surgical Studies of the Psychology Division of the Hospital das Clínicas of the University of São Paulo School of Medicine, responsible for the Diagnostic and Neuropsychology Assessment Center in São José do Rio Preto, SP, Brazil.; 2 Doctor in Clinical Psychology and Advisor of the Postgraduate Course on Sciences of the University of São Paulo School of Medicine, São Paulo, SP, Brazil.

**Keywords:** memory, three words-three shapes test, elderly, neuropsychological assessment, education, memória, idosos, avaliação neuropsicológica

## Abstract

The three words-three shapes test is a brief bedside technique for assessment of learning and memory using verbal and non-verbal material. To the best of our knowledge, performance of Brazilian elderly on this test has not yet been reported.

**Objective:**

To evaluate the performance of normal Brazilian elderly on the three words-three shapes test.

**Method:**

A total of 50 adult patients, 25 males and 25 females, with age ranging from 55 to 81 years (66.0±7.10 years), 1 to 8 years of schooling, different economic conditions and living in the São José do Rio Preto municipality, State of São Paulo, were evaluated.

**Results:**

There was no statistically significant difference between performance of males and females. Performance on incidental recall was significantly lower than in delayed recall. The performance in the learning phase improved following at least two further presentations of the stimuli. Approximately 50% of the participants did not remember the six stimuli and had to proceed to the recognition stage. The performance in the recognition stage was significantly better than during spontaneous recall. Patients with low educational level (less years of schooling) had poorer performance on the recall of shapes and on the total score of the test.

**Conclusions:**

The three words-three shapes test is rapid, efficient and straightforward to apply in the elderly, but low educational level was associated with poorer performance on this test. Normal elderly individuals had greater difficulty in the encoding process and in searching for stored information.

## Abstract (pt)

O teste três figuras-três palavras é uma técnica simples e adequada para avaliação de aprendizagem e memória verbal e não verbal. Até o presente, estudos sobre o desempenho de idosos brasileiros nesse teste não foram encontrados na literatura.

**Objetivo:**

Estudar o desempenho de idosos normais em tarefas de memória e contribuir para obtenção de dados adequados sobre o desempenho desses idosos no teste três figuras-três palavras de Mesulam.

**Método:**

Foram avaliados 50 pacientes adultos, sendo 25 do sexo masculino e 25 do feminino, com idade variando entre 55 e 81 anos (66,0±7,1 anos), 1 a 8 anos de escolaridade, diferentes situações econômicas e residentes no município de São José do Rio Preto, Estado de São Paulo.

**Resultados:**

Não foi encontrada diferença estatisticamente significativa entre o desempenho de homens e mulheres. O desempenho da memória incidental foi significativamente menor comparado ao desempenho obtido na etapa de evocação tardia. Idosos normais obtiveram melhora de desempenho com pelo menos duas novas apresentações ao estímulo. Indivíduos com menos tempo de instrução obtiveram desempenho inferior ao evocar figuras e no escore total do teste. Cerca de 50% dos participantes necessitaram realizar a etapa de reconhecimento.

**Conclusões:**

O teste três figuras-três palavras foi rápido, eficaz e de fácil aplicação para idosos. A escolaridade influenciou diretamente no desempenho do teste. Idosos normais tiveram maior dificuldade na codificação e busca da informação armazenada.

Normal development endows us with the ability to learn new information and store it in memory. Learning is a dynamic process which requires flexible behavior that draws on previous experience and is retained for life. Any event that interrupts acquisition or implies loss of previously learned material causes cognitive disorders which affect all aspects of the individual's life.

Studies focusing on the brain and main structures involved in specific tasks have increased in recent decades.^1^ This has led to study on normal individuals during the execution of activities that entail engagement of different functional systems, and has yielded more precise information on brain areas and processes involved, despite the lack of dedicated centers solely for attention, language, reasoning, perception, emotion or memory.^2^

Memory complaints are common in the elderly. Research has shown that these complaints can vary between 23% and 80%.^3^ Factors associated to these complaints include depression, anxiety, endocrine problems (hypothyroidism) and the use of certain substances.^2,6^ On the other hand, the decline of episodic memory, especially in learning tasks and free recall after intervals, has been described as an important predictive factor for Alzheimer’s disease.^4-6^

Elderly individuals do not present difficulty in memorizing of procedures. The semantic memory and short-term memory seem to be little influenced by age.^7,8^ However, age-related decline is apparent in short-term visual retention and visuospatial memory tasks.^9-11^ According to Benton,^9^ brain changes can be revealed through memory tests which employ drawing. In such tests, performance is sensitive to the effects of a brain lesion and success depends on the integrity of the retention and visual organization functions because mnemonic processes are continuous.^9,11^

In recent decades, the aims of research have focused on the study of decline of cognitive ability in the elderly population. There is a relationship between patients with subjective memory complaints and depression. Depressed elderly patients present poorer performance on memory tasks, but the executive functions present the greater impairment followed by attention deficits and reduction in processing speed^12^ Depressed patients improve with stimulus repetition, however, patients with dementia do not benefit from repetition.^13^ Depressed individuals store information and remember it after interval. The same is not true for patients presenting dementia who forget a large quantity of material after interval.^13,14^ Numerous studies have highlighted that reduced executive functions represent a differential between depressed patients and patients with dementia. Mild cognitive impairment (MCI) is the term most found in international literature to describe patients with specific compromise in mental functions but without sufficient significant alteration in daily life to characterize them as demented. The amnesic type can refer to the transition area between the normal aging process and the initial stages of Alzheimer’s disease, with a conversion rate of approximately 10% to 15% per year.^15^

This distinction between mild cognitive impairment, memory loss by treatment causes or possible dementia presentation, is a hard and delicate task that is an integral part of the routine of health professionals working in clinics and hospitals throughout Brazil. These professionals have the quantitative help of objective measures to aid differential diagnosis. Neuropsychological assessment has been cited frequently to assist in this diagnosis. Nevertheless, Brazilian neuropsychologists often use methods standardized for other cultures, or measurements which evaluate patients with high levels schooling, increasing the risk of false positives.

All these issues reinforce the need for studies on performance of the elderly Brazilian population in a bid to establish more appropriate criteria for the specific difficulties that aging can promote. Studies on instruments able to characterize clinical markers for cognitive disorders, suitable for use in our population, remain scarce in Brazil. The need to know how Brazilian individuals answer these instruments is increasing amongst health professionals.

The objective of this work was to study the performance of normal elderly on memory tasks and contribute toward the pooling of appropriate data on performance of these individuals in the three words-three shapes test.^16^

## Methods

A total of 50 adult patients, 25 males and 25 females, with age ranging from 55 to 81 years (66.0±7.10 years), 1 to 8 years of schooling, different economic conditions and living in the São José do Rio Preto municipality, State of São Paulo, were evaluated.

These individuals had no previous diagnosis of brain disease, psychiatric disorders, uncorrected visual or auditory deficit, evidence or complaints of memory loss. These data were obtained through a questionnaire devised by the examiner. This study was approved by the Research Ethics Committee of the Hospital das Clínicas of the University of São Paulo School of Medicine.

The three words-three shapes test^16^ was applied to all participants. The test comprised a sheet of paper containing stimuli of three words (pride, hungry and station) and three geometric shapes. The subject is asked to copy the three words and three shapes onto a sheet of paper without being told they are to recall these later. After copying, the sheet of paper is collected and the individual asked to draw again what they can remember from the previous photocopied items (incidental memory). If five or six stimulus are correctly reported then the test can be paused, to be resumed after thirty minutes when those individuals able to report all the stimuli are again asked which they can remember (delayed recall). However, if less than five items are recalled by the individual in the incidental memory stage, the stimuli are presented again for a further ten or fifteen seconds. In this stage, the individual is expected to study the three words and three shapes and is asked to report all the stimuli they can remember immediately afterwards (immediate memory with at least one further presentation of the stimuli). This procedure (studying words and shapes and reporting them immediately) can be repeated up to five times before interruption when the individual meets the criteria of at least five out of the six test stimuli. No further presentations of the stimuli are permitted after the fifth presentation of stimuli (length study). If the individual is unable to memorize the stimuli by the fifth try then test discontinuation is recommended.

The delayed memory is tested after thirty minutes. A blank sheet of paper is presented to the individual, on which they must reproduce the words and shapes. If spontaneous recall fails at this point, multiple choice recognition is tested (with six goals of stimulus and six false goals). The patient is instructed to circle or to strike through all the words and shapes that they are able to recall.

In the stages of incidental memory, immediate memory (with at least one exposure to stimuli) and delayed memory are attributed one point for each correct component of the figure, giving a total of five points for each correctly drawn figure, and a maximum total score of fifteen points. However, one point must be subtracted when an error occurs in reconstruction of each figure component or when the individual introduces a component which did not exist in the figure. A similar procedure is adopted for the word scoring, i.e. each correctly written word is equivalent to five points, giving a total word score of fifteen points. If gross errors in writing or addition of letters occur then a point is deducted.

The maximum achievable score for both the three figures and three words is thirty points. In the stage of recognition, five points are given for each figure and each word. A total of thirty points can be obtained if the three words and three shapes test are correctly recalled.

### Statistical analysis

Calculations were performed to evaluate associations between the variables. The Chi-square, Mann-Whitney and Pearson's correlation tests were used. For continuous variables, and more than two variables, the Kruskal-Wallis test was employed with comparison of Dunn. To correlate incidental memory and delayed memory, Spearman’s correlation test was used. All the statistical analysis was carried out using the Statistical Package software for the Social Sciences, version 10.0 (SPSS). The significance value adopted was 5%.

## Results

The 50 participants studied were distributed to give a proportion of male to female of about 50%. Of males (65.0±7.68 years; mean time of schooling=3.72±1.65 years), 80% were married and 4% widowers while in the females (67.0±6.19 years); mean time of schooling=3.80±1.89 years) 52% were married and 40% widows. The mean age of the individuals studied was 66.0±7.1 years and the mean length of schooling was 3.76±1.76 years. No statistically significant differences between male and female were observed in relation to age and schooling.

All the individuals tested copied the three words three shapes test correctly.

Comparison between males and females on the stages of incidental memory and delayed memory yielded no statistically significant difference.

In the stages of immediate memory, 44% males and 48% females needed at least one further presentation of the stimuli. The delayed memory was tested after thirty minutes.

The results obtained in the stages of incidental, immediate and delayed memory are depicted in Table 1.

**Table 1:** Score (gross) on individual stages of incidental, immediate, and delayed memory recall according to gender (N=50).

	Male				Female				Both			
	Median	Mean	SD		Median	Mean	SD		Median	Mean	SD	P
**Incidental**												
W S T	10.00 13.00 24.00	10.24 11.08 21.32	5.09 4.20 8.52		10.00 10.00 24.00	11.00 10.28 21.28	4.43 4.46 7.83		10.00 13.00 24.00	10.62 10.68 21.30	4.74 4.31 8.10	0.7621 0.4779 0.8612
**Immediate**												
W S T	15.00 14.00 28.00	13.56 13.20 26.64	2.65 2.55 4.47		15.00 14.00 27.00	13.96 13.08 27.00	1.86 2.02 3.04		15.00 14.00 28.00	13.96 13.54 27.24	1.89 2.60 2.81	0.8660 0.6539 0.9999
**Delayed**												
W S T	14.00 14.00 26.00	12.24 13.40 25.64	2.91 1.71 3.82		15.00 14.00 25.00	11.56 12.76 24.32	4.15 2.86 5.56		14.50 14.00 25.50	11.90 13.08 24.98	3.56 2.35 4.77	0.8141 0.8225 0.5405

W: words; S: shapes; T: total; SD: standard deviation; p<0.05.

There was a statistically significant difference when comparing individual performances for incidental memory and immediate memory, with delayed memory stage results (p=0.0007). The performance in incidental memory was significantly lower than (p<0.05) the result found for immediate memory (with only one further presentation of the stimulus) and the performance found in delayed memory. The performance of the immediate memory with at least two presentations of the stimuli was significantly higher than the performance found in the incidental memory stage (p<0.001). A moderate correlation was found between incidental memory and delayed memory (r_s_=0.5019).

The individuals were distributed across three age groups: 55 to 62, 63 to 70 and older than 70 years of age, where no statistically significant difference was observed among the groups. Table 2 demonstrates a comparison of mean scores for two stages of recall (incidental and delayed) in relation to age.

**Table 2:** Score gross on stages of incidental and delayed recall according to age group and gender (N=50).

	Age 55-62 (n=16)				Age 63-70 (n=20)				Age >70 (n=14)			
	Median	Mean	SD		Median	Mean	SD		Median	Mean	SD	p
**Incidental**												
W S T	10.00 14.00 24.00	10.88 10.81 21.69	4.11 4.62 7.67		15.00 13.00 27.50	12.00 11.70 23.70	4.35 3.48 7.56		9.00 8.50 18.50	8.36 9.07 17.43	5.37 4.81 8.40	0.3178 0.3624 0.0946
**Delayed**												
W S T	14.50 14.00 26.50	11.75 13.63 25.38	3.91 1.54 4.65		15.00 13.00 25.50	12.50 12.70 25.20	2.86 2.39 4.42		11.50 14.00 25.00	11.21 13.00 24.21	4.15 3.04 5.59	0.3181 0.3620 0.5966

W: words; S: shapes; T: total; SD: standard deviation.

For the analysis on available schooling, the participants were grouped according to schooling: 1 to 2, 3 to 5, and 6 to 8 years. There was a statistically significant difference in recall of figures and in total score of incidental recall (p<0.0005) between the groups 1 to 2 years and 3 to 5 years of schooling. The same difference was found when comparing the performance on recall of figures and total test performance between the groups with 1 to 2 years and 6 to 8 years of schooling (p<0.05).

There was no significant difference among the three groups of schooling regarding performance of delayed memory. The results according to schooling are shown in Table 3.

**Table 3:** Score (gross) on stages of incidental and delayed recall according to number of years of schooling (N=50).

	1 to 2 (n=8)				3 to 5 (n=35)				6 to 8 Schooling(years) (n=6)			
	Median	Mean	SD		Median	Mean	SD		Median	Mean	SD	p
**Immediate**												
W S T	7.00 5.00 12.50	7.88 6.38 14.25	3.80 4.17 4.68		12.00 13.00 24.50	10.81 11.19 22.00	4.95 3.97 8.19		15.00 14.00 29.00	13.17 13.33 26.50	2.86 2.66 5.17	0.0788 0.0168 0.0123
**Delayed**												
W S T	14.50 13.00 26.00	12.75 12.50 25.25	2.92 1.93 4.10		12.50 14.00 25.00	11.67 13.06 24.72	3.68 2.54 4.87		14.00 14.50 28.50	12.17 14.00 26.17	4.02 1.55 5.53	0.8488 0.7252 0.6422

W: words; S: shapes; T: total; SD: standard deviation.

Multiple choice recognition was applied in 48% of the males and 52% of the females whom did not achieve expected scores. In this stage, all the tested individuals correctly identified the three words and three shapes.

## Discussion

The three words-three shapes test proved a well-received and practical measure for application, since no sophisticated material is required. According to Weintraub^17^ its accessibility and ease-of-use allows application in both institutions as well as private clinics.

All the participants successfully completed the copy stage of three words and three shapes. The visual-construction ability seemed preserved in normal individuals. Nitrini et al.^18^ evaluated 30 patients with diagnosed dementia and compared the performance in these patients on neuropsychological tests versus 30 normal individuals. Among the tests employed and compared using ROC curves (receiver operator characteristics), tests of constructive abilities showed good diagnostic accuracy. In the test the individual had to copy 6 drawings, 4 of which were proposed by Rosen et al.,^19^ copy a cube and draw a house spontaneously. A number of patients and controls demonstrated significant difficulty in copying the cube picture. Among 30 patients, 29 committed more than one mistake in these pictures while no members of the control group made more than one mistake. The results found in their control group were similar to the results of the present work.

The performance of incidental memory was significantly lower than the performance found in delayed memory. These results are different to those found by Weintraub^17^ who used the three words three shapes test in 21 patients with probable diagnosis of Alzheimer’s disease, 7 patients with Korsakoff’s amnesia, and 14 control individuals. The results showed that delayed memory of words and shapes scored worse in patients with probable Alzheimer’s disease and Korsakoff’s amnesia compared with control individuals. The loss of new information over time was the best discriminator between normal elderly and subjects in the initial phase of Alzheimer’s disease.^20-24^

Our results showed that the performance of elderly people on assessment increased, provided that they were exposed to at least one further presentation of the stimuli. The performance of immediate memory following at least two further presentations of the stimuli demonstrated even greater improvement, where performance was significantly higher than that found in the incidental memory stage. This performance suggests that possible attention deficits were excluded and the elderly benefited from the stimuli repetition. These results differed to those found by Nahmias^25^ who used the same test in young adults with a high schooling level. A difference observed between our elderly sample with low schooling and the young individuals with schooling, was that the latter did not need further presentations to the stimuli.

The schooling variable influenced performance in this sample. Subjects with less schooling presented poorer performance on recall of figures and on total test performance for the incidental memory stage compared to subjects with more schooling years. In the literature, there are many studies related to the interference of schooling on cognitive test performance.^26-28^ Ardila et al.^29^ studied the performance of 806 subjects in different areas of Mexico. The participants were aged between 16 and 85 years and were divided into four groups according to schooling: illiterate, 1-4, 5-9, and more than 10 years of education, and various cognitive aspects analyzed such as attention, language, memory, visual-perception abilities, along with motor and executive functions. In general, all results were strongly associated with schooling level and it was concluded that cognitive alterations during the course of life are affected by education.

The memory deficits found with the three words three shapes test highlighted greater difficulty in the encoding (initial memory store) and retrieval (research stored material) processes. Approximately 50% of subjects in this sample needed to perform the recognition stage and 100% of these performed adequately. The elderly individuals studied in this work encountered difficulty in searching stored information, and benefited from recognition.
